# Season‐dependent impact of forage quality on stress in alpine chamois

**DOI:** 10.1002/ece3.10045

**Published:** 2023-05-01

**Authors:** Luca Corlatti, Rupert Palme, Teresa G. Valencak, Kimberlina Marie Gomez

**Affiliations:** ^1^ Chair of Wildlife Ecology and Management University of Freiburg Freiburg Germany; ^2^ Stelvio National Park – ERSAF Lombardia Bormio Italy; ^3^ Department of Biomedical Sciences University of Veterinary Medicine Vienna Austria; ^4^ College of Animal Sciences Zhejiang University Hangzhou China

**Keywords:** climate change, crude protein, diet, glucocorticoids, *Rupicapra*, stressor

## Abstract

Chronically heightened stress levels in wildlife species may have detrimental effects on individual life history traits, for example, through the increased likelihood of disease, parasitic infections, and overall reduced fitness. Understanding the drivers of stress may thus have great potential for informing wildlife conservation. Although the role of climate and individual status is well studied in stress ecology, the impact of related stressors such as dietary quality is of increasing interest to wildlife research and conservation. In this study, fecal cortisol metabolites (FCMs) in Alpine chamois *Rupicapra r. rupicapra* used as bioindicators of stress, and their relationship with forage quality—measured as the percentage of fecal crude protein (CP)—were investigated. Data collection took place in 2011 and 2012 in the Gran Paradiso National Park (Western Italian Alps), on 22 individually marked adult males. The relationship between FCMs and CPs was analyzed through linear models and separated between winter and summer months, accounting for the effect of potentially confounding exogenous and endogenous variables. After AICc‐based model selection, we found that forage quality was negatively related to FCM levels in Alpine chamois during the summer months, meaning that higher quality forage was associated with the decreased expression of stress hormones. However, during the winter months, we did not find a significant relationship, potentially as a result of forage quality being ubiquitously poor. Although the mechanisms through which dietary variations impact FCM concentrations in wildlife populations are largely unknown, the occurrence of significant relationships between forage quality and stress levels supports potentially important implications for the long‐term effect of climatic changes on the fitness of wildlife populations.

## Graphical abstract

Understanding the drivers of stress may have great potential for informing wildlife conservation. In this study, fecal cortisol metabolites were used as bioindicators of stress in chamois *Rupicapra* spp., and their relationship with forage quality was investigated. Forage quality negatively related to stress levels in chamois during the summer months, meaning that higher quality forage was associated with decreased stress levels, while in the winter months, we did not find a significant relationship, potentially as a result of forage quality being ubiquitously poor.

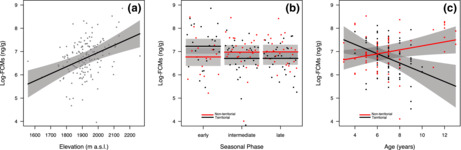

## 1 INTRODUCTION

Stress can generally be defined as a response to biological and/or environmental stimuli, also known as stressors, which may lead to physical, behavioral, and/or physiological changes (Hing et al., 2016). The stress response can impact the survival, health, and reproductive success of an individual as a result of threatening or challenging stimuli (Von Holst, 1998). Although stress is not inherently detrimental, increased chronic levels of stress can lead to negative health implications such as susceptibility to disease, parasitic infections, and overall reduced fitness (Dhabhar, 2014; Sapolsky et al., 2000). Understanding the drivers of stress may thus have great potential for informing wildlife conservation (Busch & Hayward, 2009).

Glucocorticoids (GC) are steroid hormones released by the adrenal cortex in response to stressful stimuli. They are responsible for orchestrating physiological and behavioral responses (Touma & Palme, 2005). Stress herein is the expression of GCs as a response to endo‐ or exogenous factors. The liver metabolizes circulating GCs. They are excreted via the bile into the gut (Palme et al., 2005), making analyses of fecal cortisol metabolites (FCMs) a commonly used technique to evaluate stress in wildlife studies (Palme, 2019; Sheriff et al., 2011). FCM data collection requires no handling of an animal, is non‐invasive, feedback‐free, and can provide individual‐specific data (Möstl & Palme, 2002; Sheriff et al., 2011). Accordingly, several wildlife taxa have been extensively investigated using FCMs as bioindicators of the impact of exogenous factors such as human disturbance, climate, habitat quality, and endogenous factors such as age, mating behavior, and nutrient intake on animal populations (Fattorini et al., 2018; Huber et al., 2003; Mooring et al., 2006; Rehnus et al., 2014). Hereafter, FCMs are, therefore, used as stress indicators (sensu MacDougall‐Shackleton et al., 2019).

While nutritional stress has been investigated from a quantitative standpoint, with diminished food supply being associated with increased levels of stress (e.g., Dantzer et al., 2011; Kitaysky et al., 2007; Richard Tracy et al., 2006), the impact of dietary quality on stress is relatively understudied in wildlife. It may be expected that a good quality diet should lower energetic costs and, consequently, the secretion of stress hormones. For example, fecal steroid concentrations were found to respond promptly to changes in diet composition, for example, in North American red squirrels, *Tamiasciurus hudsonicus* (Dantzer et al., 2011), or in the wild impala *Aepyceros melampus*, where increasing forage quality was associated with lower levels of fecal glucocorticoid metabolites (Hunninck et al., 2020). In particular, animal species living in environments that undergo major seasonal shifts in food availability are expected to be especially susceptible to dietary variations, but studies on free‐ranging populations are relatively rare.

The chamois *Rupicapra* spp. is the most abundant mountain ungulate in Europe and the Near East (Corlatti, Herrero, et al., 2022). It is well adapted to life in harsh environmental conditions and its dietary habits vary in response to seasonal forage availability, with forbs taken primarily in summer, whereas in autumn and winter, there is increased consumption of less nutritious Ericaceae and conifer material (Corlatti, Herrero, et al., 2022). Accordingly, the chamois alters its foraging range throughout the year in response to seasonal variations in forage availability (Corlatti, Bassano, et al., 2013). Lower‐quality habitats are generally occupied during winter months when snowfall and food availability force chamois to lower elevations (Corlatti, Herrero, et al., 2022). In recent years, several studies have been conducted using FCMs to measure the impact of several stressors on free‐living populations. These include exogenous factors such as human disturbance and climatic variables (Anderwald et al., 2021; Formenti et al., 2018; Zwijacz‐Kozica et al., 2013), as well as endogenous factors such as age and mating behavior (Corlatti et al., 2014). Previous studies investigating the impact of dietary quality, either measured through vegetation composition or fecal nitrogen content, on chamois FCM levels found somewhat contrasting results, including positive effects in the Pyrenean *R. pyrenaica pyrenaica* and Apennine *R. pyrenaica ornata* chamois (Dalmau et al., 2007; Fattorini et al., 2018), and no effect in Alpine chamois *R. rupicapra rupicapra* in the Swiss Alps (Anderwald et al., 2021). As the chamois is widely distributed and found in a range of habitats, investigations across multiple regional conditions are important to better understand its life history patterns (Corlatti, Iacolina, et al., 2022).

For this study, we focused on how forage quality related to stress levels in Alpine chamois in the Western Italian Alps. Given the importance of timescale when investigating the drivers of stress, considering that seasonal changes in FCM concentrations are to be expected in response to seasonal endocrine variations due to changes in food quality and availability (Corlatti et al., 2014; Dantzer et al., 2011), and because we wanted to ensure comparability with other studies on the same species (Anderwald et al., 2021), we focused our investigation of the relationship between FCM levels and forage quality in summer and winter, separately. We hypothesize that FCM levels would be negatively correlated with forage quality in Alpine chamois. However, due to the greater variation in vegetation quality during summer months than in winter months (Corlatti, 2020), we anticipate that the relationship between FCMs and forage quality should be stronger in the summer than in winter. Thus, during the summer months, a greater variation in forage quality would have a stronger impact on the stress response in chamois compared to the winter months.

## 2 MATERIALS AND METHODS

### 2.1 Study site

Data were collected between January 2011 and December 2012 within the Grand Paradiso National Park (GPNP) in the Western Italian Alps. The study site was a 10 km^2^ area within the upper Orco Valley (45°26′30″ N, 7°08′30″ E) between 1700 and 3000 m a.s.l., mainly southern‐exposed, and dominated by meadows of colored fescue *Festuca varia*, with patches of larch *Larix decidua* and alder shrubs *Alnus viridis*. The temperature varied between −4°C in the winter and 13°C in the summer months, with average daily precipitation between 2.8 mm in winter and 4.4 mm in autumn. Since 1922, the chamois population within the park has been protected from hunting, and at the time of the study, their densities in the study area were estimated to be around 20 individuals/km^2^ (Corlatti, Fattorini, & Nelli, 2015).

### 2.2 Data collection

Between 2010 and 2012, park personnel darted and equipped 22 adult male chamois with individual Global Positioning Systems (GPS) collars with very high frequency (VHF) beacon devices (Corlatti et al., 2012). This enabled us to detect marked males via GPS and/or VHF positioning, allowing for the identification of unique individuals and location‐specific data. In accordance with Italian law, a veterinarian was always present to provide assistance and direct observation of the animals. Further details about captures are available in Corlatti et al. (2012, 2019). As the original aim of data collection was to determine the mating behavior of adult males, female and young individuals were not monitored or sampled.

To investigate the relationship between forage quality and stress, fecal samples were collected and analyzed to determine the percentage of crude protein (CP: nitrogen content × 6.25: Robbins, 1983), as an indicator of forage quality (Gálvez‐Cerón et al., 2013; Villamuelas et al., 2017), and concentrations of fecal cortisol metabolites (FCMs), as an indicator of stress (Möstl et al., 2002). Samples were collected monthly between January 2011 and December 2012 from as many collared individuals as possible. Collared males were located using GPS data, and observed until defecation, then within 10 min, the fecal sample was collected and subsequently frozen at −20°C until laboratory analysis. The percentage of CP was determined by near‐infrared reflectance spectroscopy (NIRS): first, about 20 g of wet samples were homogenized, dried, and ground with a grinder A11 basic (Ika, Staufen, Germany). A subsample (*n* = 86, see Corlatti, Bassano, et al., 2013) was chemically analyzed for calibration to establish a standard reference for physical analysis (Nehring, 1960); the remaining samples were then analyzed using an FT‐NIR Spectrometer MPA and validated based on calibration values (details in Corlatti, 2020). FCM concentrations were analyzed with an 11‐oxoetiocholanolone enzyme immunoassay (EIA) measuring metabolites with a 5ß‐3α‐hydroxy‐11‐oxo structure (Möstl et al., 2002; validated for chamois: Anderwald et al., 2021), in 0.5 g of homogenized fecal samples, extracted and mixed with 5 mL of aqueous methanol (80%), shaken and then centrifuged (Palme et al., 2013).

To control for the effect of potential confounding variables on the relationship between CP and FCMs, the age, mating behavior (alternative reproductive tactic: ART), and environmental data at the time of sampling were accounted for. While initially sedated, the age of each animal was determined by counting horn rings (Corlatti, Gugiatti, & Imperio, 2015). ARTs were noted as either territorial (T) or non‐territorial (NT) for marked males through measuring site fidelity and intrasexual interactions, with territorial males assumed to occupy smaller home ranges and having a higher ratio of interactions won. A detailed description of the procedure used to distinguish between male types, along with full data and codes, is available in Corlatti et al. (2012, 2019). During the study period, environmental data were collected, including precipitation, snow depth, temperature, and elevation. FCM levels are excreted with a lag time thus reflecting stressors prior to collection (Huber et al., 2003; Möstl et al., 2002). Therefore, snow depth (in centimeters), minimum ambient temperature (in degrees Celsius), and cumulative precipitation (in millimeters) for the day prior to sample collection were retrieved from a meteorological station in the westernmost part of the study site (Lake Serru, 2275 m a.s.l.), while data from GPS collars provided the elevation (in meters a.s.l.) of the day before feces collection (Corlatti, 2020).

### 2.3 Statistical analysis

To investigate the relationship between CP and FCMs in different seasons, allowing for the ecological and behavioral differences between snow‐free and snow‐covered periods to be accounted for and to make our study comparable to that of Anderwald et al. (2021), data were separated into “summer” (May through October) and “winter” (November through April) months. The seasons were also separated into three bimonthly phases, “early,” “intermediate,” and “late” to further account for the unique characteristics of seasonal changes in chamois populations, such as the onset of mating behavior in early summer months (von Hardenberg et al., 2000) and the rut during early winter (early November to early December, see Corlatti et al., 2012).

All analyses were conducted with R (R Core Team, 2022) in RStudio (R Studio Team, 2022). FCMs were set as response variables, and as FCM data were not distributed homogeneously, they were log‐transformed prior to analysis (cf. Corlatti, 2018). All other variables (CP, age, ART, environmental data) were considered as potential explanatory variables and tested for multicollinearity using the variance inflation factor (all values were <3, a threshold considered to be inconsequential: Zuur et al., 2010). Pairwise chart correlations were used to determine the shape of the functional relationships between the response and explanatory variables. Two global models, that is, models including all the measured explanatory variables considered to be biologically relevant (Burnham & Anderson, 2002) were then built for the two seasons.

The global models included the predictors forage quality, precipitation, snow (only for winter), minimum temperature, elevation, and ART with interactions on both phase and age to account for the potential endo‐ and exogeneous factors influencing stress in chamois. These variables were selected because they are expected to impact on FCM levels in chamois (cf. Corlatti et al., 2014 for a rationale of the expected effects). It was expected that stress responses caused by phase and age may have been affected based on ART, while stress based on the other variables should not change by individual mating behavior. Each model also included animal identity as a random effect to account for pseudo‐replication, as the individuals were sampled at various times (Corlatti, 2018). To determine if the inclusion of the random effect improved the model, an ANOVA test was run between models with and without the random terms. As there was no significant improvement to the model with the inclusion of animal identity, a simpler model was run and animal identity was not included. The global model for the summer months was thus of the form:

$${\mathrm{FCM}}_{i}\sim N\left(\mu_{i},\sigma^{2}\right)$$

$$E\left({\mathrm{FCM}}_{i}\right)=\mu_{i}\;\text{and}\;\mathrm{var}\left({\mathrm{FCM}}_{i}\right)=\sigma^{2}$$

$$\mu_{i}={\mathrm{CP}}_{i}+{\text{precipitation}}_{i}+{\text{minT}}_{i}+{\text{elevation}}_{i}+{\text{behavior}}_{i}\times \left({\text{phase}}_{i}+{\mathrm{age}}_{i}\right)$$

While for the winter months it was of the form:

$${\mathrm{FCM}}_{i}\sim N\left(\mu_{i},\sigma^{2}\right)$$

$$E\left({\mathrm{FCM}}_{i}\right)=\mu_{i}\;\text{and}\;\mathrm{var}\left({\mathrm{FCM}}_{i}\right)=\sigma^{2}$$

$$\mu_{i}={\mathrm{CP}}_{i}+{\text{precipitation}}_{i}+{\text{snow}}_{i}+{\text{minT}}_{i}+{\text{elevation}}_{i}+{\text{behavior}}_{i}\times \left({\text{phase}}_{i}+{\mathrm{age}}_{i}\right)$$

In both models, ${\mathrm{FCM}}_{i}$ was the log‐transformed value of fecal cortisol metabolites for measure *i*, which was assumed to have a conditional normal distribution with mean $\mu_{i}$ and variance $\sigma^{2}$. The linear predictor included all the etho‐ecological variables considered important, with additive (“+”) or interactive (“×”) effects. Prior to model fitting, all continuous predictors were standardized to allow for comparability of effect size. The global models were inspected through residual plots for violation of the assumptions of linearity, homoscedasticity, and normality.

Finally, starting from the global models, a simpler structure for the summer and winter months was searched for by performing automated, all subsets model selection, where all combinations of fixed effects were compared by AICc values (Akaike Information Criterion corrected for small samples), using the the “MuMIn” package (Bartoń, 2022). Final parameter estimates were obtained by averaging all models within delta AICc <2 (Burnham & Anderson, 2002).

## 3 RESULTS

Over the 2 years, a total of 314 samples were collected from the 22 males captured (mean ± SD = 14.3 ± 5.6). For both seasons, the residual plots of the global models did not indicate a violation of model assumptions. The analyses of the summer and winter months are presented in the following sections.

### 3.1 Summer months

**FIGURE 1:**
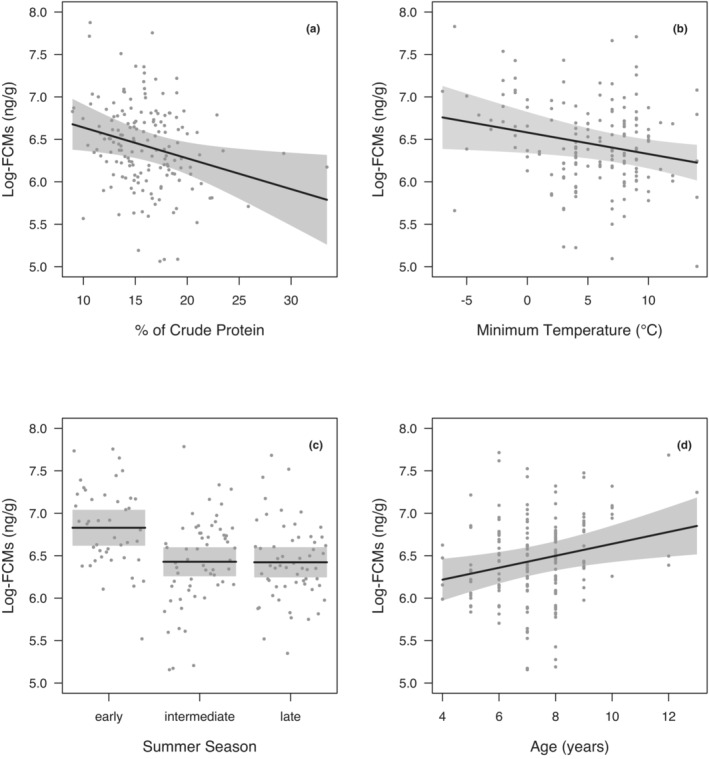
Marginal effects of the variables with a significant impact on log‐transformed FCM levels (in ng/g) in adult male Alpine chamois during summer months of 2011 and 2012 in Gran Paradiso National Park: (a) percent of crude protein, (b) minimum ambient temperature (in °C), (c) seasonal phase, and (d) age (in years).

For the summer months, the percentage of crude protein (CP), precipitation, minimum temperature, elevation, ART, seasonal phase, and age were the explanatory variables selected in the best models (Table 1). Of these, CP, minimum air temperature, seasonal phase, and age were determined to have a significant relationship with FCMs based on model averaging (Table 2). In particular, CP and minimum temperature were determined to have a negative relationship with FCMs (Table 2; Figure 1a,b). For the summer phases, FCM levels were highest during the early period compared to the intermediate and late periods, where FCM levels were similar (Table 2; Figure 1c). The age of chamois had a positive relationship with FCM levels (Table 2; Figure 1d).

**TABLE 1:** Rank of the summer models fitted to explain variation in FCM levels in Alpine chamois, within the Gran Paradiso National Park in 2011 and 2012.

CP	Prec	T_min_	Elev	ART	Phase	Age	ART:Age	Df	AICc	Delta	Weight
+	+	+		+	+	+		9	243.2	0.00	0.245
+	+	+			+	+		8	243.2	0.03	0.241
+		+			+	+		7	244.1	0.97	0.151
+		+		+	+	+		8	244.2	1.01	0.147
+	+	+		+	+	+	+	10	244.6	1.45	0.119
+	+	+	+	+	+	+		10	245.0	1.85	0.097

*Note*: Only models with delta AICc <2 are reported. The table includes the degrees of freedom (df), Akaike's information criterion corrected for small sample size (AICc), Delta AICc (Delta), and Akaike's weights (Weight). Abbreviations for predictor variables include: “CP” as a percentage of crude protein, “Prec” as precipitation, “T_min_” as minimum ambient temperature, “Elev” as elevation, “ART” as an alternative reproductive tactic, “Phase” as the seasonal phase, “Age” for the individual age.

**TABLE 2:** Average parameter estimates for summer models with delta AICc<2, selected to explain variation in log‐transformed FCM values within the Gran Paradiso National Park in 2011 and 2012.

Parameter	Estimate	SE	95LCL	95UCL
Intercept	6.929	0.100	6.734	7.124
**CP**	**−0.132**	**0.055**	**−0.240**	**−0.025**
Precipitation	−0.070	0.041	−0.150	0.009
**Minimum T**	**−0.102**	**0.049**	**−0.199**	**−0.006**
Elevation	−0.035	0.056	−0.144	0.075
ART[T]	−0.128	0.088	−0.30	0.04
**Phase[intermediate]**	**−0.411**	**0.124**	**−0.654**	**−0.168**
**Phase[late]**	**−0.411**	**0.134**	**−0.673**	**−0.150**
**Age**	**0.102**	**0.044**	**0.016**	**0.188**
ART[T]:Age	0.074	0.085	−0.091	0.240

*Note*: The table reports parameter estimates with associated standard error (SE) and lower and upper bounds of 95% confidence interval (95LCL and 95UCL, respectively). Statistically significant estimates in bold.

### 3.2 Winter months

**FIGURE 2:**
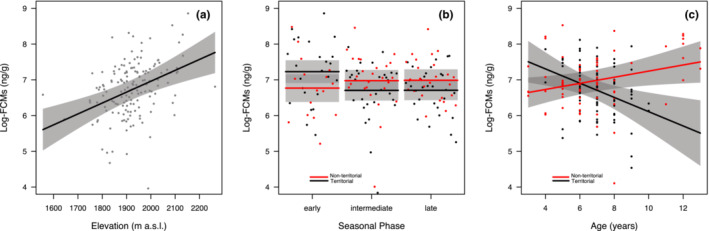
Marginal effects of the variables with a significant impact on log‐transformed FCM levels (in ng/g) in adult male Alpine chamois during winter months of 2011 and 2012 in Gran Paradiso National Park: (a) elevation (in m a.s.l.), (b) seasonal phase, and (c) age (in years) based on mating behavior (territorial and non‐territorial).

For the winter months, the percentage of crude protein (CP), precipitation, elevation, ART, seasonal phase, age, and the interactive effect of ART with phase and age were the explanatory variables selected in the best models (Table 3). Of these, elevation and the interaction of mating behavior with phase and age were determined to have a significant relationship with FCMs based on model averaging (Table 4). In particular, elevation was determined to have a positive relationship with FCMs (Table 4; Figure 2a). The influences of phase and age on FCM levels were observed to differ based on mating behavior. During the intermediate and late winter phases, territorial males exhibited lower levels of FCM compared to non‐territorial males (Table 4; Figure 2b). For age by behavior, territorial males had a negative relationship between age and FCM levels, while non‐territorial males had a positive association between age and FCM levels (Table 4; Figure 2c).

**TABLE 3:** Rank of the winter models fitted to explain variation in FCM levels in Alpine chamois, within the Gran Paradiso National Park in 2011 and 2012. Only models with delta AICc < 2 are reported.

CP	Prec	Elev	ART	Phase	Age	ART:Phase	ART:Age	Df	AICc	Delta	Weight
		+	+		+		+	6	367.1	0.00	0.295
		+	+	+	+	+	+	10	367.6	0.55	0.224
+		+	+		+		+	7	367.7	0.67	0.211
+		+	+	+	+	+	+	11	368.6	1.53	0.137
	+	+	+		+		+	7	368.7	1.60	0.132

*Note*: The table includes the degrees of freedom (df), Akaike's information criterion corrected for small sample size (AICc), Delta AICc (Delta), and Akaike's weights (Weight). Abbreviations for predictor variables include: “CP” as percentage of crude protein, “Prec” as precipitation, “Elev” as elevation, “ART” as alternative reproductive tactic, “Phase” as the seasonal phase, “Age” for the individual age.

**TABLE 4:** Average parameter estimates for winter models with delta AICc < 2, selected to explain variation in log‐transformed FCM values within the Gran Paradiso National Park in 2011 and 2012.

Parameter	Estimate	SE	95LCL	95UCL
Intercept	6.887	0.162	6.569	7.204
CP	0.085	0.073	−0.058	0.228
Precipitation	−0.049	0.065	−0.177	0.079
**Elevation**	**0.290**	**0.076**	**0.141**	**0.439**
ART[T]	0.132	0.318	−0.492	0.755
Phase[intermediate]	0.213	0.249	−0.275	0.701
Phase[late]	0.198	0.253	−0.298	0.693
Age	0.148	0.082	−0.014	0.310
**ART[T]:Phase[intermediate]**	**−0.719**	**0.333**	**−1.371**	**−0.067**
**ART[T]:Phase[late]**	**−0.728**	**0.333**	**−1.381**	**−0.075**
**ART[T]:Age**	**−0.491**	**0.156**	**−0.797**	**−0.184**

*Note*: The table reports parameter estimates with associated standard error (SE) and lower and upper bounds of 95% confidence interval (95LCL and 95UCL, respectively). Statistically significant estimates in bold.

## 4 DISCUSSION

Forage quality during the summer months was negatively associated with FCM levels in male Alpine chamois. Over the same period, the age of the individual, the phase of the season, and the mean temperature were all significantly associated with FCM levels. During the winter months, FCM levels were not significantly associated with forage quality, while they were significantly associated with elevation, as well as with age, and phase of the season based on mating behavior.

Previous studies on the relationship between dietary quality and stress have generally concluded that a higher‐quality diet is associated with decreased stress levels. In crop‐raiding Asian elephants *Elephas maximus*, wild grizzly *Ursus arctos horribilis*, and black *Ursus americanus* bears, a higher‐quality diet led to lower FCM levels, even when exposed to additional stressors such as human‐induced threats (Pokharel et al., 2019; Stetz et al., 2013). Similarly, decreased levels of FCMs were associated with a higher‐quality diet in red deer *Cervus elaphus* (Anderwald et al., 2021) and a higher‐quality habitat in roe deer *Capreolus capreolus* (Horcajada‐Sánchez et al., 2019). However, increased glucocorticoid levels were recorded in white‐tailed deer *Odocoileus virginianus* fawns fed on energetically richer diets (Taillon & Côté, 2008). Forage digestibility should be an important factor for ungulates with characteristics intermediate between mixed feeders and browsers; accordingly, it is expected that chamois would respond to lower forage quality in all seasons (cf. Anderwald et al., 2021). In fact, in Pyrenean and Apennine chamois, forage quality was found to negatively influence FCM concentrations (Dalmau et al., 2007; Fattorini et al., 2018). Conversely, Anderwald et al. (2021) concluded that, in their study area, there was no correlation between fecal nitrogen and FCM concentrations in Alpine chamois, neither in summer nor in the winter months.

Our findings support the existence of a significant relationship between dietary quality and FCMs only in summer months, where increasing percentages of crude protein were associated with decreased levels of FCMs. Contrary to our hypothesis, forage quality during winter months was not significantly associated with FCMs in Alpine chamois.

During winter, the body condition of individuals may have deteriorated to the extent that glucocorticoids could be suppressed to prevent further loss of fat reserves (cf. Anderwald et al., 2021). Additionally, in Alpine environments, forage quality in the winter months is generally ubiquitously poor, and the little variation in crude protein observed in our study site in this period (see Corlatti, 2020), reduces the possibility to detect significant relationships. When compared to the results of Anderwald et al. (2021), unknown ecological differences between study sites might contribute to explaining the contrasting patterns in the summer months. Additionally, the study of Anderwald et al. (2021) was partly biased toward female chamois (roughly 76% of the study population), while our study was performed in males: it cannot be excluded that the contrasting results were caused by sex‐specific stress responses, for example, related to sex‐specific hormones that impact glucocorticoid secretion and excretion, possibly by different receptors that lead to distinct physiological responses (cf. Lalande et al., 2022).

The mechanisms underlying the relationship between dietary quality and stress remain speculative, though they may involve the effects of fiber on fecal mass, gut passage time, biliary excretion of steroids, or changes in steroid hormone metabolite structure following variation in microbial activity (Dantzer et al., 2011). As gut bacteria can influence levels of FCM concentrations through metabolism and hormone secretion (Vogt et al., 2023), changes to diet composition may alter these microbiota. Understanding how specific diets influence the microbiome of the intestinal tract can further illuminate the reason how diet influences hormone expression, specifically, whether it influences GC levels in Alpine chamois. In our study, we were not concerned with gut passage time, as previous studies on bovids have noted variations related to pre‐ and post‐weaning stages (Vogt et al., 2023), and our study included only adult individuals.

More generally, it should be stressed that a multitude of factors may influence FCM levels, including variations in environmental conditions and social status (Nelson, 1999). In our study, several potentially confounding variables were accounted for, though we cannot exclude the occurrence of unmeasured variables that may contribute to explain the observed pattern. In summer, the negative effect of minimum ambient temperature on stress is consistent with previous studies that further concluded lower temperatures resulted in higher stress levels in Alpine chamois (Anderwald et al., 2021; Corlatti et al., 2014). It should be noted that extremely high temperatures did not occur during the study period, and therefore it cannot be definitely stated that inceased temperatures would lead to lower stress levels, but rather that favorable ranges of temperatures decrease stress levels in chamois. The higher FCM levels during early summer months compared to mid and late summer were consistent with previous studies (Anderwald et al., 2021), and may be explained by the onset of mating behavior in Alpine chamois occurring during late spring, here early summer, potentially leading to elevated stress levels relating to male territoriality (Corlatti et al., 2019; von Hardenberg et al., 2000). Lastly, the positive association between age and FCM in summer was consistent with what was observed for chimpanzees *Pan troglodytes* (Emery Thompson et al., 2020) and gray mouse lemur *Microcebus murinus* (Hämäläinen et al., 2015). However, the underlying mechanisms in our study species remain unclear: while aging is associated with a dysfunction in the regulation of the hypothalamic–pituitary–adrenal axis, resulting in an overproduction of glucocorticoids (Emery Thompson et al., 2020), our sampled males could not be considered as senescent or old males.

During the winter months, elevation had a positive relationship with FCM values. As snow depth was obtained from measurements from a weather station, and not spatially explicit, elevation may be considered a proxy for snow depth: accordingly, as male chamois tend to occupy lower elevations due to a thinner snow cover, the observed pattern may relate to resource availability. The winter phase based on mating tactics was also determined to have an impact on FCM levels. As the rut occurs in November, territorial males have been observed to increase their mating efforts and display higher levels of aggression and dominance compared to non‐territorial males, potentially driving increased levels of stress (Corlatti et al., 2012; Corlatti, Caroli, et al., 2013). During the post‐rut, non‐territorial males have been observed to interact more, as territorial males end their mating effort and females may still be receptive (Corlatti et al., 2012): consequently, this may lead to increased stress compared to territorial males during the intermediate and later winter periods. Lastly, age based on mating tactics also influences FCM levels in winter. One potential explanation for this is that young males experience elevated stress levels while trying to defend (or intrude into) territories. As territorial males become more experienced, the stress of maintaining their territory may decrease, while the stress of seeking and competing for potential mates in non‐territorial males does not diminish with experience.

Increased stress levels of wild animals can have potentially detrimental impacts on the fitness of populations, an understanding of what leads to heightened FCMs concentrations is critical for conservation (Busch & Hayward, 2009). As food resources are key to mountain ungulate population dynamics (Lovari et al., 2020), by altering the vegetation dynamics in mountain environments, climate change and increasing temperature may ultimately affect the nutritional quality and phenology of plants. It is well known that several ecological factors can influence vegetation quality (Albon & Langvatn, 1992; Van Soest, 1994): for example, it has recently been observed that increasing levels of CO_2_ decrease forage nitrogen concentrations (Dumont et al., 2015), and this, in turn, may influence dietary quality in free‐ranging species. By incorporating FCMs as physiological biomarkers of dietary quality into this pattern, future studies may reveal the indirect relationships among climatic variables, forage quality, stress level, and life history traits such as growth, reproduction, and survival, which should improve our understanding of the dynamics of wildlife populations.

## AUTHOR CONTRIBUTIONS

**Luca Corlatti:** Conceptualization (equal); data curation (equal); formal analysis (equal); investigation (lead); methodology (lead); writing – original draft (equal). **Rupert Palme:** Conceptualization (supporting); data curation (equal); writing – original draft (supporting); writing – review and editing (equal). **Teresa Valencak:** Conceptualization (supporting); data curation (equal); writing – original draft (supporting); writing – review and editing (equal). **Kimberlina Marie Gomez:** Conceptualization (equal); data curation (equal); formal analysis (equal); investigation (supporting); methodology (supporting); writing – original draft (equal).

## CONFLICT OF INTEREST STATEMENT

We have no competing interests.

## Data Availability

Data used for analysis are available at: https://doi.org/10.5061/dryad.931zcrjqz.

## References

[ece310045-bib-0001] Albon, S. D. , & Langvatn, R. (1992). Plant phenology and the benefits of migration in a temperate ungulate. Oikos, 65, 502–513.

[ece310045-bib-0002] Anderwald, P. , Andri, S. C. , & Palme, R. (2021). Reflections of ecological differences? Stress responses of sympatric alpine chamois and red deer to weather, forage quality, and human disturbance. Ecology and Evolution, 11, 15740–15753. 34824786 10.1002/ece3.8235 PMC8601901

[ece310045-bib-0003] Bartoń, K. (2022). MuMIn: Multi‐model inference. R package version 1.46.0. http://CRAN.R‐project.org/package=MuMIn

[ece310045-bib-0004] Burnham, K. P. , & Anderson, D. R. (2002). Model selection and multimodel inference. Springer.

[ece310045-bib-0005] Busch, D. S. , & Hayward, L. S. (2009). Stress in a conservation context: A discussion of glucocorticoid actions and how levels change with conservation‐relevant variables. Biological Conseervation, 142, 2844–2853.

[ece310045-bib-0006] Corlatti, L. (2018). Fecal cortisol metabolites under anonymized sampling: Robust estimates despite significant individual heterogeneity. Ecological Indicators, 95, 775–780.

[ece310045-bib-0007] Corlatti, L. (2020). Anonymous faecal sampling and NIRS studies of diet quality: Problem or opportunity? Ecology and Evolution, 10, 6089–6096. 32607215 10.1002/ece3.6354 PMC7319235

[ece310045-bib-0008] Corlatti, L. , Bassano, B. , Valencak, T. G. , & Lovari, S. (2013). Foraging strategies of male chamois. Journal of Zoology, 291, 111–118.

[ece310045-bib-0009] Corlatti, L. , Béthaz, S. , von Hardenberg, A. , Bassano, B. , Palme, R. , & Lovari, S. (2012). Hormones, parasites and male mating tactics in alpine chamois: Identifying the mechanisms of life history trade‐offs. Animal Behaviour, 84, 1061–1070.

[ece310045-bib-0010] Corlatti, L. , Caroli, M. , Pietrocini, V. , & Lovari, S. (2013). Rutting behaviour of territorial and nonterritorial male chamois: Is there a home advantage? Behavioural Processes, 92, 118–124. 23182914 10.1016/j.beproc.2012.11.008

[ece310045-bib-0011] Corlatti, L. , Fattorini, L. , & Nelli, L. (2015). The use of block‐counts, mark‐resight and distance sampling to estimate population size of a mountain‐dwelling ungulate. Population Ecology, 57, 409–419.

[ece310045-bib-0012] Corlatti, L. , Gugiatti, A. , & Imperio, S. (2015). Horn growth patterns in alpine chamois. Zoology, 118, 213–219. 25869383 10.1016/j.zool.2015.01.003

[ece310045-bib-0013] Corlatti, L. , Herrero, J. , Ferretti, F. , Anderwald, P. , García‐González, R. , Hammer, S. , Nores, C. , Rossi, L. , & Lovari, S. (2022). Northern chamois *Rupicapra rupicapra* (Linnaeus, 1758) and southern chamois *Rupicapra pyrenaica* Bonaparte, 1845. In L. Corlatti & F. Zachos (Eds.), Terrestrial Cetartiodactyla – Handbook of the mammals of Europe (pp. 51–86). Springer Nature.

[ece310045-bib-0014] Corlatti, L. , Iacolina, L. , Safner, T. , Apollonio, M. , Buzan, E. , Ferretti, F. , Hammer, S. E. , Herrero, J. , Rossi, L. , Serrano, E. , Arnal, M. C. , Brivio, F. , Chirichella, R. , Cotza, A. , Crestanello, B. , Espunyes, J. , Fernández de Luco, D. , Friedrich, S. , Gačić, D. , … Šprem, N. (2022). Past, present and future of chamois science. Wildlife Biology, 4, e01025.

[ece310045-bib-0015] Corlatti, L. , Lorenzetti, C. , & Bassano, B. (2019). Parasitism and alternative reproductive tactics in northern chamois. Ecology and Evolution, 9, 8749–8758. 31410277 10.1002/ece3.5427 PMC6686307

[ece310045-bib-0016] Corlatti, L. , Palme, R. , & Lovari, S. (2014). Physiological response to etho‐ecological stressors in male alpine chamois: Timescale matters! The Science of Nature, 101, 577–586. 10.1007/s00114-014-1195-x 24908399

[ece310045-bib-0017] Dalmau, A. , Ferret, A. , Chacon, G. , & Manteca, X. (2007). Seasonal changes in fecal cortisol metabolites in Pyrenean chamois. Journal of Wildlife Management, 71, 190–194.

[ece310045-bib-0018] Dantzer, B. , McAdam, A. G. , Palme, R. , Boutin, S. , & Boonstra, R. (2011). How does diet affect fecal steroid hormone metabolite concentrations? An experimental examination in red squirrels. General and Comparative Endocrinology, 174, 124–131. 21871893 10.1016/j.ygcen.2011.08.010

[ece310045-bib-0019] Dhabhar, F. S. (2014). Effects of stress on immune function: The good, the bad, and the beautiful. Immunologic Research, 58, 193–210. 24798553 10.1007/s12026-014-8517-0

[ece310045-bib-0020] Dumont, B. , Andueza, D. , Niderkorn, V. , Lüscher, A. , Porqueddu, C. , & Picon‐Cochard, C. (2015). A meta‐analysis of climate change effects on forage quality in grasslands: Specificities of mountain and Mediterranean areas. Grass and Forage Science, 70, 239–254.

[ece310045-bib-0021] Emery Thompson, M. , Fox, S. A. , Berghänel, A. , Sabbi, K. H. , Phillips‐Garcia, S. , Enigk, D. K. , Otali, E. , Machanda, Z. P. , Wrangham, R. W. , & Muller, M. N. (2020). Wild chimpanzees exhibit humanlike aging of glucocorticoid regulation. Proceedings of the National Academy of Sciences of the United States of America, 117, 8424–8430. 32229565 10.1073/pnas.1920593117 PMC7165472

[ece310045-bib-0022] Fattorini, N. , Brunetti, C. , Baruzzi, C. , Macchi, E. , Pagliarella, M. C. , Pallari, N. , Lovari, S. , & Ferretti, F. (2018). Being “hangry”: Food depletion and its cascading effects on social behaviour. Biological Journal of the Linnean Society, 125, 640–656.

[ece310045-bib-0023] Formenti, N. , Viganó, R. , Fraquelli, C. , Trogu, T. , Bonfanti, M. , Lanfranchi, P. , Palme, R. , & Ferrari, N. (2018). Increased hormonal stress response of Apennine chamois induced by interspecific interactions and anthropogenic disturbance. European Journal of Wildlife Research, 64, 68.

[ece310045-bib-0024] Gálvez‐Cerón, A. , Serrano, E. , Bartolomé, J. , Mentaberre, G. , Fernández‐ Aguilar, X. , Fernández‐Sirera, L. , Navarro‐González, N. , Gassó, D. , López‐Olvera, J. R. , Lavín, S. , Marco, I. , & Albanell, E. (2013). Predicting seasonal and spatial variations in diet quality of Pyrenean chamois (*Rupicapra pyrenaica pyrenaica*) using near infrared reflectance spectroscopy. European Journal of Wildlife Research, 59, 115–121.

[ece310045-bib-0025] Hämäläinen, A. , Heistermann, M. , & Kraus, C. (2015). The stress of growing old: Sex‐ and season‐specific effects of age on allostatic load in wild grey mouse lemurs. Oecologia, 178, 1063–1075. 25847061 10.1007/s00442-015-3297-3

[ece310045-bib-0026] Hing, S. , Narayan, E. , Thompson, R. C. , & Godfrey, S. (2016). The relationship between physiological stress and wildlife disease: Consequences for health and conservation. Wildlife Research, 43, 51–60.

[ece310045-bib-0027] Horcajada‐Sánchez, F. , Escribano‐Ávila, G. , Lara‐Romero, C. , Virgós, E. , & Barja, I. (2019). The effect of livestock on the physiological condition of roe deer (*Capreolus capreolus*) is modulated by habitat quality. Scientific Reports, 9, 15953. 31685886 10.1038/s41598-019-52290-7 PMC6828671

[ece310045-bib-0028] Huber, S. , Palme, R. , & Arnold, W. (2003). Effects of season, sex, and sample collection on concentrations of fecal cortisol metabolites in red deer (*Cervus elaphus*). General and Comparative Endocrinology, 130, 48–54. 12535624 10.1016/s0016-6480(02)00535-x

[ece310045-bib-0029] Hunninck, L. , May, R. , Jackson, C. R. , Palme, R. , Røskaft, E. , & Sheriff, M. J. (2020). Consequences of climate‐induced vegetation changes exceed those of human disturbance for wild impala in the Serengeti ecosystem. Conservation Physiology, 8, coz117. 32477568 10.1093/conphys/coz117 PMC7246078

[ece310045-bib-0030] Kitaysky, A. S. , Piatt, J. F. , & Wingfield, J. C. (2007). Stress hormones link food availability and population processes in seabirds. Marine Ecology Progress Series, 352, 245–258.

[ece310045-bib-0031] Lalande, L. , Gilot‐Fromont, E. , Carbillet, J. , Débias, F. , Duhayer, J. , Gaillard, J. M. , Lemaître, J. F. , Palme, R. , Pardonnet, S. , Pellerin, M. , Rey, B. , & Vuarun, P. (2022). The environmental roots of the relationship between glucocorticoids and body mass in the wild. *bioRxiv* . 10.1101/2022.09.15.508076

[ece310045-bib-0032] Lovari, S. , Franceschi, S. , Chiatante, G. , Fattorini, L. , Fattorini, N. , & Ferretti, F. (2020). Climatic changes and the fate of mountain herbivores. Climatic Change, 162, 2319–2337.

[ece310045-bib-0033] MacDougall‐Shackleton, S. A. , Bonier, F. , Romero, L. M. , & Moore, I. T. (2019). Glucocorticoids and “stress” are not synonymous. Integrative Organismal Biology, 1, obz017. 33791532 10.1093/iob/obz017 PMC7671118

[ece310045-bib-0034] Mooring, M. S. , Patton, M. L. , Lance, V. A. , Hall, B. M. , Schaad, E. W. , Fetter, G. A. , Fortin, S. S. , & McPeak, K. M. (2006). Glucocorticoids of bison bulls in relation to social status. Hormones and Behavior, 49, 369–375. 16257404 10.1016/j.yhbeh.2005.08.008

[ece310045-bib-0035] Möstl, E. , Maggs, J. , Schrötter, G. , Besenfelder, U. , & Palme, R. (2002). Measurement of cortisol metabolites in faeces of ruminants. Veterinary Research Communications, 26, 127–139. 11922482 10.1023/a:1014095618125

[ece310045-bib-0036] Möstl, E. , & Palme, R. (2002). Hormones as indicators of stress. Domestic Animal Endocrinology, 23, 67–74. 12142227 10.1016/s0739-7240(02)00146-7

[ece310045-bib-0037] Nehring, K. (1960). Agrikulturchemische Untersuchungsmethoden für Dünge‐ und Futtermittel, Böden und Milch. Parey.

[ece310045-bib-0038] Nelson, R. J. (1999). An introduction to behavioral endocrinology. Sinauer.

[ece310045-bib-0039] Palme, R. (2019). Non‐invasive measurement of glucocorticoids: Advances and problems. Physiology & Behavior, 199, 229–243. 30468744 10.1016/j.physbeh.2018.11.021

[ece310045-bib-0040] Palme, R. , Rettenbacher, S. , Touma, C. , El‐Bahr, S. M. , & Möstl, E. (2005). Stress hormones in mammals and birds: Comparative aspects regarding metabolism, excretion, and noninvasive measurement in fecal samples. Annals of the New York Academy of Sciences, 1040, 162–171. 15891021 10.1196/annals.1327.021

[ece310045-bib-0041] Palme, R. , Touma, C. , Arias, N. , Dominchin, M. F. , & Lepschy, M. (2013). Steroid extraction: Get the best out of fecal samples. Wiener Tierarztliche Monatsschrift‐Veterinary Medicine Austria, 100, 238–246.

[ece310045-bib-0042] Pokharel, S. S. , Singh, B. , Seshagiri, P. B. , & Sukumar, R. (2019). Lower levels of glucocorticoids in crop‐raiders: Diet quality as a potential ‘pacifier’ against stress in free‐ranging Asian elephants in a human‐production habitat. Animal Conservation, 22, 177–188.

[ece310045-bib-0043] R Core Team . (2022). R: A language and environment for statistical computing. R Foundation for Statistical Computing. https://www.R‐project.org/

[ece310045-bib-0044] Rehnus, M. , Wehrle, M. , & Palme, R. (2014). Mountain hares (*Lepus timidus*) and tourism: Stress events and reactions. Journal of Applied Ecology, 51, 6–12.

[ece310045-bib-0045] Richard Tracy, C. , Nussear, K. E. , Esque, T. C. , Dean‐Bradley, K. , Tracy, C. R. , DeFalco, L. A. , Castle, K. T. , Zimmerman, L. C. , Espinoza, R. E. , & Barber, A. M. (2006). The importance of physiological ecology in conservation biology. Integrative and Comparative Biology, 46, 1191–1205. 21672817 10.1093/icb/icl054

[ece310045-bib-0046] Robbins, C. T. (1983). Wildlife feeding and nutrition. Academic Press Inc.

[ece310045-bib-0047] R Studio Team . (2022). RStudio: Integrated development for R. RStudio Inc.

[ece310045-bib-0048] Sapolsky, R. , Romero, L. M. , & Munck, A. (2000). How do glucocorticoids influence stress responses? Integrating permissive, suppressive, stimulatory, and preparative actions. Endocrine Reviews, 21, 55–89. 10696570 10.1210/edrv.21.1.0389

[ece310045-bib-0049] Sheriff, M. J. , Dantzer, B. , Delehanty, B. , Palme, R. , & Boonstra, R. (2011). Measuring stress in wildlife: Techniques for quantifying glucocorticoids. Oecologia, 166, 869–887. 21344254 10.1007/s00442-011-1943-y

[ece310045-bib-0050] Stetz, J. , Hunt, K. , Kendall, K. C. , & Wasser, S. K. (2013). Effects of exposure, diet, and thermoregulation on fecal glucocorticoid measures in wild bears. PLoS One, 8, e55967. 23457488 10.1371/journal.pone.0055967 PMC3573068

[ece310045-bib-0051] Taillon, J. , & Côté, S. D. (2008). Are faecal hormone levels linked to winter progression, diet quality and social rank in young ungulates? An experiment with white‐tailed deer (*Odocoileus virginianus*) fawns? Behavioral Ecology and Sociobiology, 62, 1591–1600.

[ece310045-bib-0052] Touma, C. , & Palme, R. (2005). Measuring fecal glucocorticoid metabolites in mammals and birds: The importances of validations. Annals of the New York Academy of Sciences, 1046, 54–74. 16055843 10.1196/annals.1343.006

[ece310045-bib-0053] Van Soest, P. J. (1994). Nutritional ecology of the ruminant. Cornell University Press.

[ece310045-bib-0054] Villamuelas, M. , Serrano, E. , Espunyes, J. , Fernández, N. , López‐Olvera, J. R. , Garel, M. , Santos, J. , Parra‐Aguado, M. Á. , Ramanzin, M. , Fernández‐Aguilar, X. , Colom‐Cadena, A. , Marco, I. , Lavín, S. , Bartolomé, J. , & Albanell, E. (2017). Predicting herbivore faecal nitrogen using a multispecies near‐infrared reflectance spectroscopy calibration. PLoS One, 12, e0176635. 28453544 10.1371/journal.pone.0176635 PMC5409079

[ece310045-bib-0055] Vogt, A. , König von Borstel, U. , Waiblinger, S. , Palme, R. , & Barth, K. (2023). Fecal cortisol metabolites reflect transport stress in three‐month old dairy calves pre‐ and post‐weaning – A pilot study. Journal of Dairy Science (in press), 106, 2124–2136. 36631319 10.3168/jds.2022-22341

[ece310045-bib-0056] von Hardenberg, A. , Bassano, B. , Peracino, A. , & Lovari, S. (2000). Male alpine chamois occupy territories at hotspots before the mating season. Ethology, 106, 617–630.

[ece310045-bib-0057] Von Holst, D. (1998). The concept of stress and its relevance for animal behavior. Advances in the Study of Behavior, 27, 1–131.

[ece310045-bib-0058] Zuur, A. , Leno, E. , & Elphick, C. (2010). A protocol for data exploration to avoid common statistical problems. Methods in Ecology and Evolution, 1, 3–14.

[ece310045-bib-0059] Zwijacz‐Kozica, T. , Selva, N. , Barja, I. , Silván, G. , Martínez‐Fernández, L. , Illera, J. C. , & Jodłowski, M. (2013). Concentration of fecal cortisol metabolites in chamois in relation to tourist pressure in Tatra National Park (South Poland). Acta Theriologica, 58, 215–222.

